# Targeting HTLV-1 Activation of NFκB in Mouse Models and ATLL Patients

**DOI:** 10.3390/v3060886

**Published:** 2011-06-21

**Authors:** Daniel A. Rauch, Lee Ratner

**Affiliations:** Department of Medicine, Division of Molecular Oncology, Washington University School of Medicine, Campus Box 8069, 660 S. Euclid Ave., St. Louis, MO 63110, USA; E-Mail: drauch@dom.wustl.edu

**Keywords:** HTLV-1, tax, NFκB, mouse models, ATLL therapy

## Abstract

Of the millions of HTLV-1 infected carriers worldwide, 3–5% will develop an aggressive T-cell neoplasm that is highly refractory to conventional therapy. The virus carries the Tax oncogene which constitutively activates the NFκB pathway. This co-option of signaling through NFκB provides for the HTLV-1 infected cell an escape from cell cycle arrest and apoptosis, a steady source of growth factors, and a mechanism by which the virus can activate its own target cell. Therapies that target the NFκB pathway sensitize adult T-cell leukemia/lymphoma (ATLL) cells to apoptosis. A focus on translational interrogation of NFκB inhibitors in animal models and ATLL patients is needed to advance NFκB-targeted ATLL therapies to the bedside.

## 1. Introduction

Of the 15–20 million HTLV-1 infected carriers worldwide, more than 500,000 will develop an aggressive T-cell neoplasm that is highly refractory to conventional therapy. Despite intensive efforts to improve the overall survival, adult T-cell leukemia/lymphoma (ATLL) remains one of the hematologic malignancies with the poorest prognosis. ATLL is typically preceded by decades of clinical latency during which infected cells accumulate selectable traits leading to malignant transformation. Host pathways commandeered by the virus can be used as therapeutic targets and a constitutively activated NFκB pathway has emerged as an essential hit in the development of ATLL. Research into the mechanisms underlying HTLV-1 modulation of the NFκB pathway has revealed the extent to which HTLV-1 oncogenesis is dependent on the NFκB pathway [1,2]. Mouse models of ATLL have also been created to recapitulate the virus induced, NFκB-dependent leukemias and lymphomas. Translational investigations of NFκB inhibitors in mouse models and ATLL patients are necessary to bring this work from bench to bedside.

## 2. HTLV-1 Oncogenesis Depends on NFκB

**Figure 1.**
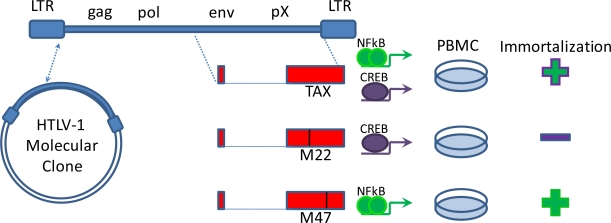
Tax activation of NFκB is required for immortalization. The creation of a molecular clone of HTLV-1 by Kimata *et al.* [11] enabled the analysis of Tax mutants for immortalization determinants [12]. Mutations that inhibit Tax activation of the NFκB pathway prevented immortalization of peripheral blood mononuclear cells (PBMC), whereas mutations that inhibit Tax activation of the CREB pathway allowed NFκB activation and Tax-mediated immortalization.

During decades of clinical latency HTLV-1 modulates host signaling pathways to promote proliferation of infected cells. Among the genes carried in the viral genome, the 40 kDa viral transcriptional transactivator (Tax) is sufficient to promote cellular transformation. The mechanism utilized by Tax to promote cellular transformation is multi-faceted, broadly involving activation of proliferation, dysregulation of cell cycle checkpoints, and promotion of genetic instability. While the affects of Tax expression in a cell are diverse, Tax depends on a central signaling pathway for its transforming activity. Mouse models in which Tax is expressed in the lymphocyte compartment have constitutively active NFκB and reproduce many aspects of HTLV-1 pathogenesis (Table 1). Tax expression in the absence of other viral factors is sufficient to activate the NFκB pathway and cause leukemia, lymphoma, solid tumors, splenomegaly, and osteolytic disease. More than 20 years ago Nerenberg identified a role for NFκB in tumor growth in a transgenic mouse model in which Tax, under the regulation of the HTLV-1 LTR, developed neurofibromas [3]. However, LTR-driven Tax expression in mouse models most often resulted in disorders associated with chronic inflammation, another NFκB-mediated process [4–7]. Other promoters were used in transgenic constructs to restrict Tax expression to the lymphoid compartment and better model ATLL-like malignancies. In one mouse model, the granzyme B promoter was used to drive Tax expression in activated T-cells and NK cells. These mice developed leukemia and lymphoma and tumors associated with high levels of NFκB and NFκB-regulated genes [8]. Tax driven by the Lck promoter also causes an ATLL-like malignancy in transgenic mice in which NFκB is constitutively elevated [9,10]. The necessity of NFκB in Tax-mediated transformation was confirmed using HTLV-1 molecular clones in which a single point mutation in the Tax oncogene that disrupts its ability to activate the NFκB pathway (Figure 1) also eliminates the virus’ ability to transform primary cells [11,12]. These data establish that constitutive activation of the NFκB pathway is essential in the process of Tax-mediated oncogenesis.

## 3. The NFκB Signaling Pathway

**Figure 2.**
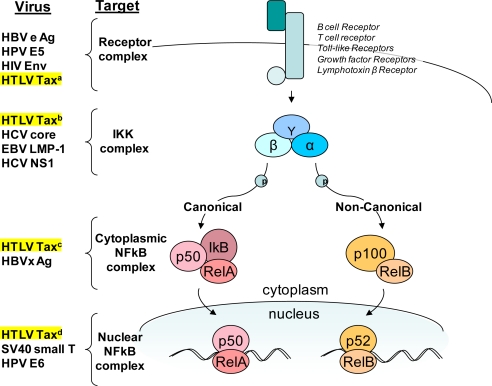
Tax targets the NFκB pathway. A simplified schematic of NFκB signaling pathways highlighting regulatory proteins commonly targeted by viruses [1,2,13]. HTLV-1 Tax targets the pathway at several points. (**a**) Tax leads to the activation of receptor-associated kinases that signal through the NFκB pathway [16,17]. (**b**) Tax directly binds to IKKγ (NEMO) which leads to constitutive phosphorylation and degradation of the NFκB repressor IκB [15]. (**c**) Tax activates and recruits IKKα to p100 stimulating phosphorylation, ubiquitination, and processing to p52 leading to nuclear translocation [19]. (**d**) Tax alters binding or recognition of a variety of transcription factors and DNA binding proteins increasing the number of genes regulated by the NFκB pathway [18].

The NFκB family of transcription activators are involved in many biological processes including cell survival, stress responses, and development [13]. NFκB is also a central regulator of immune effectors including cytokine and chemokine secretion, receptor expression, antigen presentation, cellular proliferation, and programmed cell death. The NFκB family contains five members (RelA, RelB, cRel, p50, and p52) that can form 15 different homo or heterodimers with a variety of activities and tissue specificities. Latent or unstimulated cells retain NFκB proteins in the cytoplasm bound to various inhibitors of kappa B (IκB) proteins. Upon activation, IκB kinases (IKKs) release NFκB complexes which then translocate to the nucleus through two distinct pathways, termed canonical and non-canonical. These arms are distinct but retain extensive mechanisms of cross-regulation [13]. The canonical pathway, which is activated by pro-inflammatory signals, is IKKγ dependent, while the non-canonical pathway functions independent of IKKγ, instead depending on NFκB inducing kinase (NIK). The non-canonical pathway is activated in response to tumor necrosis factor (TNF) receptors during lymphoid development. It is because NFκB proteins regulate these myriad pathways that cancers and viral pathogens exploit NFκB to enhance proliferation, cell survival, and evasion of immune responses (Figure 2). Consequently, this array of human cancers could also all be targeted by NFκB directed therapies.

## 4. Tax Targets the NFκB Pathway

Lymphoma is commonly associated with constitutive NFκB activity and oncogenic human viruses, including Epstein-Barr virus (EBV), Human papillomavirus (HPV), Hepatitis B virus (HBV), and Hepatitis C virus (HCV) all carry viral factors that directly target and co-opt the NFκB pathway during oncogenesis. HTLV-1 uses Tax in multiple strategies to efficiently activate both the canonical and non-canonical NFκB pathways (Figure 2).

Tax directly interacts with several NFκB members, including RelA, p50, p52, IKKγ, and the NFκB precursor protein p100 such that both arms of the NFκB pathway are involved in tumorigenesis [1,14]. Rapid and reversible, the canonical pathway of NFκB is transient and strictly regulated. Tax activates the canonical pathway through a variety of mechanisms including binding to IKKγ, activating the Akt pathway which signals through IKKα, or by promoting phosphorylaton of p65 via activation of pro-inflammatory cytokines [15–18]. HTLV-1 uses the canonical pathway, through Bcl-xl, to prevent intrinsic apoptosis [20]. The non-canonical arm of NFκB, which involves new protein synthesis of p100 and RelB downstream of NIK, is slower and irreversible. Tax-induced p100 processing or its physical interaction with Tax in the nucleus contribute to Tax activation of the non-canonical NFκB pathway as well. The non-canonical pathway has a broader anti-apoptotic effect on both apoptotic pathways through BCL-XL, FLIP, XIAP, and CIAP, making it a more powerful therapeutic target [20]. In fact, the difference in transforming potential between HTLV-1 and HTLV-2 has been linked to the ability of HTLV-1 Tax to activate the non-canonical arm of the NFκB pathway [21].

## 5. NFκB and Apoptosis

Constitutive activation of NFκB family members and downstream effectors provides several selective advantages to malignant cells. One selective advantage offered by unregulated NFκB signaling is the ability to bypass checkpoint controls and p53-mediated apoptosis. In transformed cell lines as well as freshly isolated ATLL samples, NFκB activation supports the survival and proliferation of HTLV-1 infected cells. Moreover, lymphoma cell lines with constitutively activated NFκB are resistant to a variety of inducers of apoptosis including gamma-irradiation, etoposide, and combinations of cycloheximide and TNF or TRAIL, and resist the activation of both the intrinsic and extrinsic apoptotic pathways [20]. Like ATLL cells, malignant cells arising in mouse models are also resistant to chemotherapy and radiation-induced apoptosis [22]. Although mutations deleting or inactivating p53 are common in ATLL, Tax is capable of bypassing p53-dependent cell-cycle checkpoints through constitutive activation of the NFκB pathway [22]. Tax is known to suppress a wide range of pro-apoptotic factors and stimulate expression of factors acting as apoptosis inhibitors [14]. The necessity of NFκB in Tax-mediated resistance to apoptosis is revealed when overexpression of IκB or loss of p65 represses the NFκB pathway, and re-sensitizes Tax-transformed cells to inducers of p53-mediated apoptosis [23].

## 6. NFκB and Inflammation

**Figure 3.**
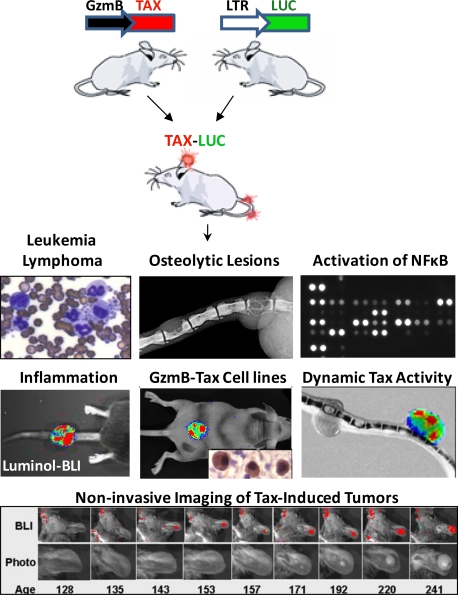
Tax transgenic mice model ATLL. Several Tax transgenic mouse models of ATLL have demonstrated the sufficiency of Tax as an independent oncogene. Second generation strains, such as the one depicted, have added capabilities, which enable non-invasive interrogation of various Tax activities using bioluminescence imaging [30,31,35].

HTLV-1 in humans is also associated with chronic inflammation which is mediated by NFκB. Chronic inflammation resulting from Tax-mediated canonical NFκB activity leads to HAM/TSP in some HTLV-1 carriers [24], but may also contribute to development of ATLL. Chronic inflammation promotes cancer through complex mechanisms involving cytokine mediated proliferation, stromal activation, immune modulation, and release of DNA damage promoting agents [25,26]. Genes activated by Tax in transgenic mice are directly or indirectly regulated by NFκB inducible cytokines that promote inflammation and immune cell infiltration (Figure 3). Tax tumor cells express IL-6, M-CSF, IL-1, TNF-α, and Tax expression enhances IL-6 and TNF-α expression *in vitro* and *in vivo* [27,28]. Moreover, malignant cells express NFκB inducible cytokines and stimulate cytokine production in tumor infiltrating cells and stroma. Factors produced by Tax-tumor cells cause splenomegaly, neutrophilia, elevated white count and anemia in transgenic mice and SCID recipients of tumor allografts [27,29]. Preceding tumorigenesis in transgenic mice, Tax and the NFκB pathway promote a state of chronic inflammation in which Tax-induced malignancies can thrive [30].

This inflammation-associated malignancy is Tax-dependent and promoter-dependent since SV40 large T under the same promoter does not reproduce the inflammation promoting nature of Tax tumors [29,31]. Interestingly, chronic inflammation is a complicating factor because it carries both tumor-promoting and tumor-repressing effects. IFNγ, an NFκB inducible gene found in chronic inflammation, causes inhibition of tumor angiogenesis and represses tumor growth [32]. The mechanism by which Tax promotes inflammation and tumorigenesis is coupled by its regulation of the NFκB pathway.

## 7. NFκB and T-Cells

HTLV-1 infects and transforms CD4^+^ T-cells. The NFκB pathway is essential for T-cell functions including T-cell development, activation, gene expression, cell cycle progression, survival, cytokine production, and apoptosis [13]. While Tax activates NFκB in T-cells, most freshly isolated ATLL cells do not express detectable levels of Tax [33]. This has been explained by the discovery that Tax is a primary target of cytotoxic T lymphocyte (CTL) attack, and HTLV-1 infected T-cells that express high levels of Tax are destroyed [34]. Is ATLL Tax-independent at the time of clinical presentation? A mouse model in which Tax drives expression of firefly-luciferase, allowed non-invasive, real-time detection of Tax activity using bioluminescence imaging (Figure 3). This model revealed that Tax activity, which was normally undetectable, was occasionally punctuated by short bursts of intense expression. Moreover, these stochastic bursts of Tax expression preceded advancing stages of tumorigenesis [31]. In addition, a triple-transgenic mouse strain carrying an ovalbumin-inducible T-cell receptor (TCR) transgene demonstrated that systemic T-cell activation accelerated the development of Tax-induced lymphoma [35]. Determining whether ATLL ever attains Tax independence awaits an inducible-Tax mouse in which Tax expression can be repressed late in malignancy. Importantly, in ATLL cells the NFκB pathway remains activated when Tax expression is repressed. Thus NFκB remains a therapeutic target even when Tax is not expressed.

## 8. Targeting NFκB *in vivo*

NFκB regulates the expression of a wide variety of genes implicated in proliferation, angiogenesis, invasion, and metastasis and the dependence of HTLV-1 oncogenesis on the NFκB pathway makes it an ideal target for therapeutic attack. Repression of the NFκB pathway could make ATLL cells sensitive to apoptosis, slow their proliferation, or repress aspects of the immune response that promote malignancy. In tissue culture and mouse models, non-specific inhibitors of the NFκB pathway like sodium salicylate or cyclopentenone prostaglandins can increase the sensitivity of Tax-tumor cells to apoptosis and repress NFκB-inducible cytokines IL-6, IL-10, IL-15, and IFN-γ [28]. Bortezomib is another non-specific inhibitor of the NFκB pathway that is capable of inhibiting proliferation of Tax tumors cells *ex vivo* and sensitizing cells to apoptosis [36]. Bortezomib treatment slowed tumor growth in an allograft model by increasing apoptosis, but toxicity constraints limited the efficacy of the treatment [36]. Bay11-7082, an IKK inhibitor, inhibits the NFκB pathway in ATLL cells and sensitizes HTLV-1 infected cells lines as well as primary ATLL cells to apoptosis [37]. Over the past six years several additional studies have therapeutically targeted the NFκB pathway in order to kill ATLL cells [38]. Oridonin, NIK-333, curcumin, fucoidan, histone-deacetylase inhibitors, and a derivative of epoxyquinomicin C have all been reported to induce apoptosis in ATLL cells by repressing the NFκB pathway [39–44]. These findings serve as sufficient proof of principle that NFκB-targeted therapies show great promise against ATLL. The field now awaits successful clinical trials *in vivo*.

## 9. Targeting NFκB in ATLL Patients

**Figure 4.**
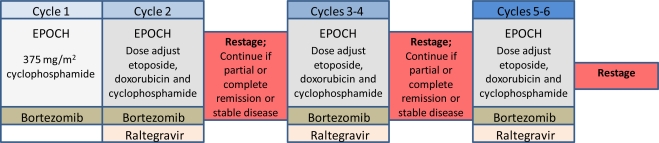
Schema for an ATLL clinical trial using NFκB targeted therapy. The clinical trial shown has been approved and is currently accepting patients. Clinical trials of NFκB-targeted combination chemotherapies are now beginning to apply the information obtained from primary research to clinical practice.

The majority of ATLL patients present with acute or lymphomatous ATLL, which results in a median survival of 0.5–2.0 years, despite intensive chemotherapy treatment [45,46]. To determine if NFκB blockade is tolerated in these patients, and whether or not it improves response rates and overall survival, our current multicenter trial combines infusional chemotherapy (EPOCH) with bortezomib (Figure 4). In addition, this clinical trial includes treatment with integrase inhibitor raltegravir, which was found to inhibit HTLV-1 integration in tissue culture [47]. The addition of an antiviral agent to this ATLL treatment regimen is based on our previous clinical trial in which chemotherapy was found to markedly enhance virus expression in a subset of patients [48].

## 10. Conclusions

Host pathways can be used as chemotherapeutic targets when they confer an essential trait to the cancer cell. A constitutively activated NFκB pathway represents such a target in the case of HTLV-1 mediated ATLL. The NFκB pathway provides an escape from cell cycle arrest and apoptosis, a steady source of growth factors, and a mechanism by which the virus can activate its own target cell. Accumulating evidence supports the concept that NFκB targeted therapies sensitize ATLL cells to apoptosis. Research in ATLL therapies should now focus on translational interrogation of NFκB inhibitors in animal models and ATLL patients.

## Figures and Tables

**Table 1.** Summary of tax transgenic mouse models of adult T-cell leukemia/lymphoma (ATLL).

**Promoter**	**Gene**	**Strain**	**Diseases Observed**	**Relevance to ATLL**	**Merits of Model**	**Weaknesses of Model**	**Therapies Tested**	**Ref**
HTLV LTR	Tax	C57BL/6 X DBA/2 X CD1	Mesenchymal Tumors Thymic atrophy	Not Observed	Overexpression of TGF-beta, activation of NFκB	Tax transforms fibroblasts but not thymocytes	NFκB ODN	[49] [50] [51]
			Neurofibromatosis Adrenal Tumors	Not Observed	Tax activation of Nerve growth factor and repression of NF1	Not associated with HTLV associated diseases in humans	None	[52] [53] [54] [55]
			Muscle degeneration	Myositis	High levels of Tax expression in muscle	Incomplete penetrance	None	[56]
			Exocrinopathy Lymphadenopathy Splenomegaly	Sjogren syndrome ocular lesions	NfκB inflammatory disorders associated with Tax	Caused by B not T lymphocytes	None	[57] [58]
			Bone Turnover	Lytic bone lesions	NFkB associated bone lesions	Incomplete penetrance	None	[59]
	Tax βgal		Mesenchymal Tumors	Not Observed	Tax expression in response to tissue damage	Tissue damage not correlated with tumor	None	[60] [61]
HTLV LTR	pX	C57BL/6 X CD1	Thymic Atrophy	Not Observed	Effects of pX on thymus independent of promoter used	pX gene expression not detectable	None	[62]
Ig-SV40								
MMTV LTR								
HTLV LTR	pX	C3H/HeN	Inflammatory Arthropathy Osteogenesis Autoimmunity	Arthritis	IL-1, IL-6, TNFα, TGFβ detected in joints.	No malignancy	Anti-Fas mAb (RK-8)	[4] [5] [7] [63] [64]
	Tax							
CD4	Tax							
HTLV LTR	Tax	C3H	Mesenchymal Tumors	NFκB mediated malignancy	IκB degradation leads to constitutive NFκB activation	Expression restricted to CNS and testes	None	[65]
Ig	Tax	FVB/N	Not Observed	Not Observed	Lymphoma with CNS involvement	Roles of c-Myc and Tax unclear	None	[66]
Ig HTLV LTR	Tax c-myc		CD4+ Lymphoma CNS Tumors	CD4^+^ Lymphoma				
GzmB	Tax	C57BL/6	LGL lymphoma Leukemia Osteolytic lesions Splenomegaly Lymphadenopathy Hypercalcemia	Lymphoma Leukemia Lytic bone lesions Hypercalcemia	NFkB mediated leukemia lymphoma	Not a CD4+ T cell malignancy	Bortezomib	[8] [36]
	Tax IL-2−/−				IL-2 not required for phenotype	Not a CD4+ T cell malignancy	None	[29]
	Tax IFNγ−/−				Accelerated tumor onset and death	May also affect tumor immunity	None	[32]
	Tax P53−/−				Accelerated disease progression	Only seen in P53+/− mice	None	[22]
GzmB ApoE	Tax OPG				Reduced cancer and bone lesions	Causes osteopetrosis	Zoledronic Acid	[27]
GzmB HTLV LTR	Tax LUC	C57BL/6 X FVB			Bioluminescent tumors	Not a CD4+ T cell malignancy	None	[31]
GzmB HTLV LTR	Tax LUC ARF−/−		Lymphoma Bone Turnover Osteosarcoma		ARF−/− is not equivalent to p53−/−	Osteosarcoma not associated with ATLL	Zoledronic Acid	[67]
GzmB HTLV LTR TCR	Tax LUC TCR^ova^	C57BL/6 X FVB X BALB/c	Leukemia Lymphoma Lymphadenopathy		Tax induced by wounding and T cell activation leads to enhanced tumorigenesis	Primary malignancy not a not a CD4+ T cell malignancy	None	[35]
EμSRα TET TET	tTA Tax M47	FVB/N	Alopecia Hyperkeratosis Splenomegaly	Skin Lesions	Tet-inducible model allows repression of Tax	No malignancy	None	[68]
EμSRα TET	tTA M22		Not observed	Not Observed	Control establishes role of NFκB in disease			
Lck-prox	Tax	C57BL/6	CD4− CD25+ pre-T cell Leukemia Lymphoma	Leukemia Lymphoma	Cancer stem cells derived from these mice recapitulate disease in SCID	Not a CD4+ T cell malignancy	As_2_O_3_ + IFN-α AMD3100	[9] [69] [70] [71]
Lck-dis	Tax	C57BL/6 X DBA/2	CD4+ CD25− mature T cell leukemia lymphoma Arthritis	Leukemia Lymphoma	Mature CD4+ or CD8+ T cell malignancy	Cells lack CD25	None	[72] [73]
CD3-ɛ	Tax	C57BL/6 X CBA	Mesencymal tumors Mammary Adenoma	Not Observed	Tax associated with apoptosis and p53	Not a CD4+ T cell malignancy	None	[74]
